# Corrigendum to Factors associated with COVID-19 length of hospitalization and mortality during four epidemic waves, March 2020–November 2021, Suriname

**DOI:** 10.26633/RPSP.2023.139

**Published:** 2023-09-08

The *Pan American Journal of Public Health* draws readers’ attention to an error in the following article, pointed out by the authors:

Byline and suggested citation in page 1 last name of co-author should read Jarbandhan instead of Jarbanha

